# Hyaluronic Acid/Chitosan/Glycerophosphate-Based In Situ-Forming Hydrogel for Accelerated Wound Healing

**DOI:** 10.3390/gels11100835

**Published:** 2025-10-17

**Authors:** Hadeia Mashaqbeh, Rania Hamed, Hiba Alzoubi, Rana Obaidat, Mohammad Alnaeif, Meriem Rezigue, Hala T. Abukassab, Wasan Al-Farhan, Mohammad Obeid

**Affiliations:** 1 Pharmaceutics and Pharmaceutical Technology Department, Faculty of Pharmacy, Yarmouk University, Irbid 21163, Jordan; meriem.rezigue@yu.edu.jo (M.R.);; 2 Department of Pharmacy, Faculty of Pharmacy, Al-Zaytoonah University of Jordan, P.O. Box 130, Amman 11733, Jordanwasanmortada@gmail.com (W.A.-F.); 3 Department of Basic Pathological Sciences, Faculty of Medicine, Yarmouk University, Irbid 21163, Jordan; 4 Department of Pharmaceutics and Pharmaceutical Technology, Faculty of Pharmacy, The University of Jordan, Amman 11733, Jordan; 5 Pharmaceutical and Chemical Engineering Department, School of Applied Medical Sciences, German Jordanian University, Amman Madaba Street, P.O. Box 35247, Amman 11180, Jordan; mohammad.alnaief@gju.edu.jo

**Keywords:** injectable gel, thermoresponsive, wound healing, combined therapy

## Abstract

This study reports the use of an in situ-forming gel based on hyaluronic–chitosan–glycerophosphate for wound healing. Hydrogels with optimized thermoresponsive gelling, rheological, and prolonged drug release properties were developed and incorporated with ciprofloxacin and carvacrol. In vitro evaluations included rheological studies, swelling degree, degradation rates, morphological analysis, antioxidant effects, antimicrobial activity, and drug release studies. The effectiveness of the optimized hydrogel was assessed using an animal ischemic wound rabbit ear model. The incorporation of ciprofloxacin and carvacrol into the combined hydrogel system maintained the mechanical strength of the formula, with a G′/G″ ≈ ratio of approximately 15.6, interconnected porosity, and controlled swelling. It enhanced antimicrobial activity against both *S. aureus* and *E. coli*. In addition, the developed gel exhibited sustained release following the Higuchi diffusion kinetics. The quantitative wound area% indicated that on day 9, the mean wound area decreased from 81.8% for the control to 51.2% for the developed gel. The study findings demonstrate the suitability and potential of this system as multifunctional wound-healing formulations that promote moist healing, antimicrobial and antioxidant activities, while providing sustained therapeutic delivery over 24 h.

## 1. Introduction

Biopolymers have gained popularity in the last decade as a “green” alternative to synthetic polymeric materials. Even though they cannot wholly replace synthetic polymers, biopolymers should be optimized to capitalize on their low toxicity and biodegradability [1].

Hyaluronic acid (HA) has been extensively studied in the biomaterial field and is utilized in the preparation of hydrogels for various clinical applications [2]. HA is a linear polysaccharide comprising repeated disaccharide units of N-acetyl-D-glucosamine and D-glucuronic acid attached by alternating β-1,4 and β-1,3 glycosidic bonds [3]. Fabricating hydrogels that contain pure HA is challenging due to their high cost, low mechanical strength, and inadequate injectability [4,5]. This drives the need for alternate compositions to formulate effective HA-based hydrogels.

Researchers have employed combined polymers (hybrid polymers) to prepare hydrogels that are prepared with two or more polymers and are crosslinked, making it difficult to easily disassemble and form promising, stable HA-based hydrogels [6,7].

Chitosan is a biopolymer produced by the alkaline deacetylation of its precursor chitin. In nature, chitin is the most common biopolymer after cellulose. Chitin is found in the cell walls of fungi and the exoskeletons of crustaceans. The source and preparation procedure of chitosan determine its physicochemical characteristics, such as molecular weight and deacetylation degree, which are related to the proportion of glucosamine units, as well as the distribution of acetylglucosamine and glucosamine units. Chitosan is playing an increasingly important role in the biomedical field due to its abundance, biocompatibility, biodegradability, bioadhesiveness, penetration enhancement properties, and bacteriostatic effects. Chitosan has been incorporated into various formulations and dosage forms, including tablets, capsules, films, beads, microparticles, microspheres, liquid gels, textile fibers, and sponges [8,9,10]. In these preparations, the crosslinking of the chitosan increases its mechanical properties, which is achieved either by covalent chemical crosslinking or noncovalent physical binding. The physical crosslinking to form the gel is obtained by simple physical mixing of constituents under the appropriate conditions [11,12,13].

Glycerophosphate, a naturally occurring organic compound, has been approved by the FDA for the treatment of unbalanced phosphate metabolism through intravenous administration [11]. In a study by Chenite et al. [9], a chitosan thermo-responsive gel system was reported for the first time, where they prepared a promising thermosensitive neutral injectable gel-forming solution containing chitosan and glycerophosphate [14,15]. BioSyntech (Laval, QC, Canada) registered this technique under the name BST-Gel [16].

Developing chitosan and hyaluronic acid hydrogel using electrostatic crosslinking without any chemical crosslinking or modifications is highly attractive as part of the green formulation approach. These hydrogels reduce potential toxicity and environmental concerns while maintaining biocompatibility and biodegradability.

The incorporation of hyaluronic acid into chitosan-hydrogel is expected to enhance the hydrogel’s mechanical properties, microstructure, pore sizes, and release properties due to the interactions of hyaluronic carboxyl and hydroxyl groups with chitosan amino groups, thus avoiding premature gel degradation and burst drug release [17]. In addition to the pointed role of hyaluronic acid in the wound healing process, as it promotes cell migration, tissue regeneration, and proliferation, these factors make chitosan-hyaluronic-based systems an emerging candidate for wound healing use. However, to achieve injectability and ease of application, the reported hyaluronic-chitosan-based hydrogels for wound dressings usually involve chemical modifications of both hyaluronic acid and chitosan [18,19,20]. Therefore, the investigation of hyaluronic–chitosan–glycerophosphate in situ-forming gel will gain the advantage of injectability, as it can be easily applied as a liquid and fit the irregular geometries of the wound, then converted to gel to provide better coverage and drug release properties.

Herein, we report the use of hyaluronic acid/chitosan/glycerophosphate-based hydrogel for wound healing. This hydrogel was previously designed for the delivery of anticancer drugs with in situ gelation at 37 °C. This study presented the optimization and in vitro and in vivo evaluation of this hydrogel system for wound healing applications, which can prolong drug release with an enhanced antimicrobial effect. The in situ gelling conditions were adjusted to 33 °C to be more relevant to wound treatment. Furthermore, the therapeutic effectiveness of the formulations was enhanced by the combined incorporation of two model drugs: ciprofloxacin, a broad-spectrum antibacterial agent, and carvacrol, a natural compound with both antioxidant and antibacterial properties. In vitro evaluations, including rheological studies, swelling and degradation rates by gravimetric analysis, and hydrogel morphology using scanning electron microscopy (SEM), were performed. In addition to other physicochemical characterizations, antioxidant effects, antimicrobial activity, drug release studies, and release kinetics were investigated. Moreover, the effectiveness of this hydrogel formulation was assessed through an in vivo wound healing animal study using an ischemic wound rabbit ear model, demonstrating its promising potential as a multifunctional wound dressing that combines antibacterial and antioxidant properties, along with extended drug release, suitable rheological characteristics, and an in situ gelling forming ability.

## 2. Results and Discussion

### 2.1. Preparation of Chitosan-Hyaluronic Acid-Glycerophosphate In Situ-Forming Hydrogels

Chitosan hydrogel (F0) was prepared using in situ thermo-responsive gelation of chitosan polymer. This system remained a viscous liquid at low temperatures, and the hydrogel formed as the temperature increased. Anionic β-glycerophosphate (GP) was used for ionic crosslinking of cationic chitosan polymer. The formation of hydrogel is attributed to electrostatic and hydrophobic interactions between chitosan chains, in addition to the electrostatic interactions between chitosan cationic ammonium groups and the anionic phosphate groups of GP [21]. A homogenous gel was formed within 3 min of maintaining the chitosan-GP mixture in a thermostatic water bath using the tube inversion method. Hybrid CS/HA/GP hydrogels (F1-F3) were prepared by combining chitosan and hyaluronic polymers in the presence of β-glycerophosphate. All hydrogels were thermo-responsive and able to form a complete gel within 3 min (Section 2.2.4); thus, loading the hydrogel with ciprofloxacin did not affect the gelation time. Meanwhile, adding carvacrol (F1-cip-Crv) results in a slight increase in gel formation time. However, this difference cannot be considered statistically significant, as the *p*-value is greater than 0.05.

### 2.2. Physicochemical Characterization of Hydrogels

#### 2.2.1. FTIR Analysis

**Figure 1:**
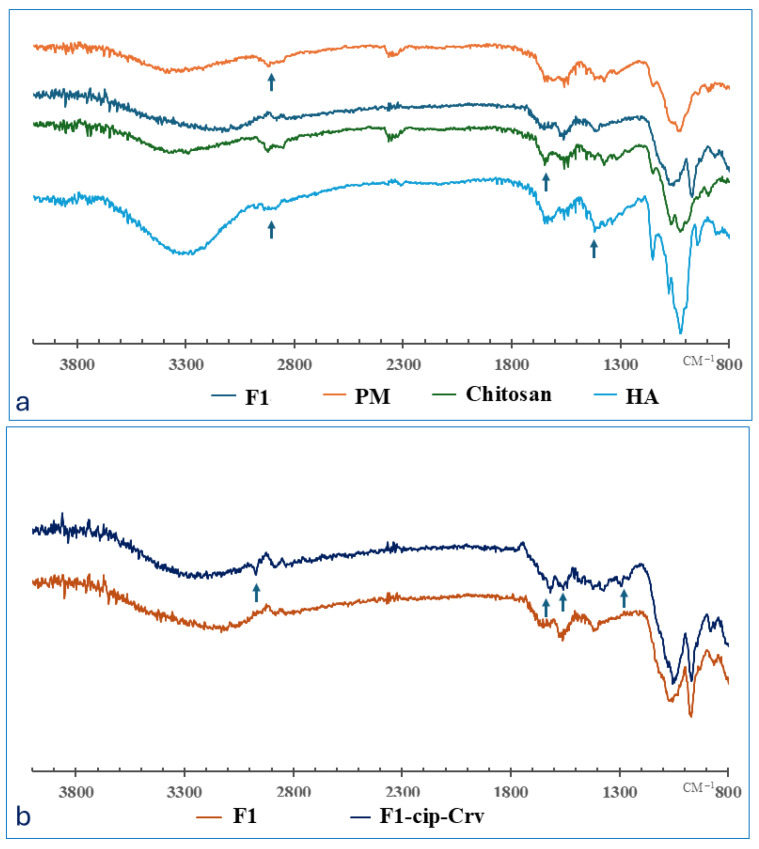
FTIR spectrum of (**a**) raw polymers, physical mixture (PM), blank gel (F1), and (**b**) blank gel compared to the drug-loaded hydrogel (F1-cip-Crv): the arrows indicate the main bands of drugs and polymers.

To explore the interactions concerning the structure of raw CS, HA, drug, drug-loaded, and physical mixtures of blinded raw polymers (PM) and blank hydrogels, FTIR analysis was conducted (Figure 1). Their FTIR spectra shows the characteristic functional groups of the raw materials (Table S1). The IR spectrum of HA (Figure 1a) shows a distinct peak at 1031 cm^−1^ related to C-O-C stretching, another band at 1620 cm^−1^ associated with the asymmetrical C=O stretching of COO−, and 1417 cm^−1^ attributed to the symmetrical C=O stretching of COO−. In addition, the peaks between 3323 and 3550 cm^−1^ were assigned to the OH groups [22].

Compared to raw polymers and physical mixtures, the FTIR spectra of the formed hydrogel displayed a shift of the broad O–H and N–H stretching peaks (3200–3500 cm^−1^) toward the lower range, which suggests the formation of hydrogen bonding between HA, CS, and Gp. In addition, a decrease in the intensity of amid I (1650 cm^−1^) and amid II (1556 cm^−1^) can be attributed to electrostatic interactions. The introduction of a new phosphate peak at 974 cm^−1^ confirms the incorporation of glycerophosphate.

FTIR spectra of drug-loaded hydrogel (F1-Cip-Crv) showed the existence of ciprofloxacin characteristic peaks beside hyaluronic and chitosan polymer characteristic peaks (Figure 1b), e.g., 1267 cm^−1^ and 1050 cm^−1^ corresponding to C–O stretching vibrations and C-F groups of ciprofloxacin, respectively. Peaks around 1500 and 1600 cm^−1^ indicate the existence of a carvacrol aromatic structure. The characteristic peaks of ciprofloxacin and carvacrol are present in the loaded hydrogel, with no significant deviation, indicating the compatibility and incorporation of ciprofloxacin with the chitosan and hyaluronic acid polymers.

#### 2.2.2. SEM Analysis

**Figure 2:**
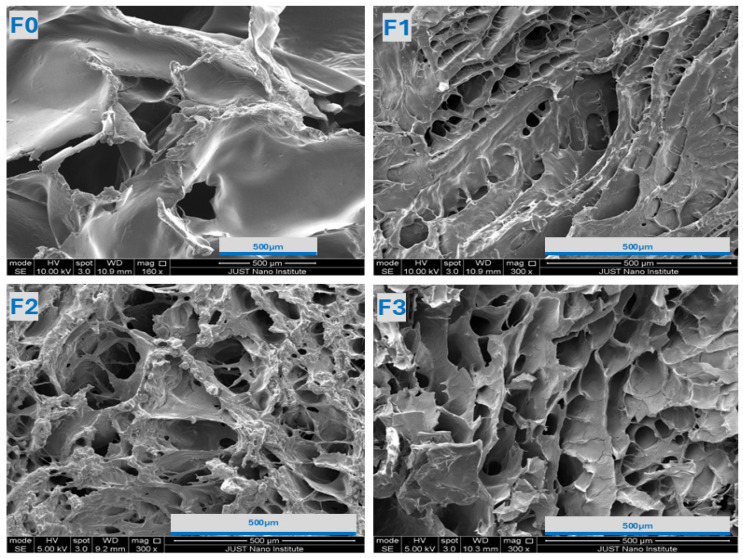
Scanning Electron Microscopy images of (**F0**–**F3**) hydrogels, with magnifications of 150× (**F0**) and 300× (**F1**–**F3**), scale bar = 500 µm.

To gain additional insight into the hydrogel’s morphological structure, topographical images at different magnifications were taken using SEM and are shown in Figure 2. The observed well-defined, interconnected porous network structure indicates the presence of polymeric crosslinking. Compared to the chitosan-GP hydrogel (F0), the addition of HA into the hydrogel strengthens the network, resulting in a smaller, more porous structure with a denser network. This can be attributed to the electrostatic interactions between HA and CS. The further addition of HA leads to an increase in a more open network, which can be justified by the excess HA containing hydrophilic functional groups that can hold more water and swell, resulting in larger pore sizes.

The absence of drug-related structures, such as distinct particles or crystal domains, on the surface suggests that the drug is incorporated or molecularly dissolved within the polymeric structures. Morphological analysis was performed on the lyophilized hydrogel formulations to evaluate the internal microstructures of the gel, and quantitative pore size distribution measurements would be valuable for future investigations.

#### 2.2.3. Swelling and In Vitro Degradation Studies

**Figure 3:**
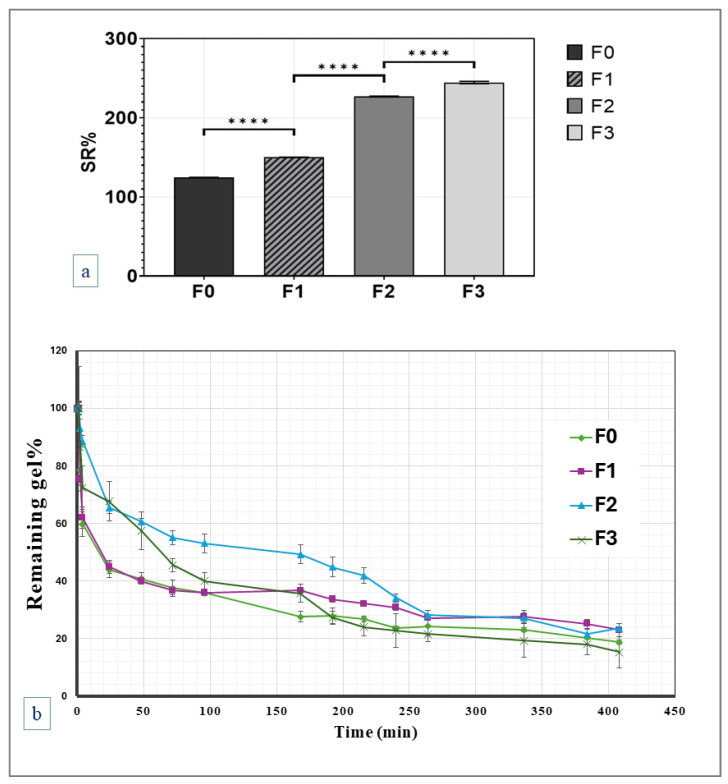
(**a**) Equilibrium swelling ratios of studied hydrogels (F0-F3) and (**b**) remaining percentages of hydrogel over time (*n* = 3). **** *p* value < 0.0001.

The equilibrium swelling and in vitro degradation of F0-F3 coded-hydrogels were calculated, as shown in Figure 3. Lower swelling ratios at low HA concentrations result from the dense and compact network’s restriction of water intake. Higher HA concentrations lead to a more porous and open structure, which enhances water absorption and, consequently, swelling ratios. This behavior correlates with the observed changes in pore size and network compactness as determined by the SEM analysis. In addition, the swelling study results showed that the developed gel formulations can maintain their structural integrity for four hours after reswelling, before structural loss and gradual degradation begin. This suggests the hydrogels can maintain their porosity and structure during the initial reswelling stage.

Swelling, combined with slow polymer degradation, accounts for the slow-release rate of the drug from the developed hydrogels. The extent and rate of swelling mainly depend on the hydrogel composition and cross-linking. Figure 3 presents the average weight change in evaluated hydrogel formulations, reflecting the hydrogel’s swelling and degradation characteristics.

Several mechanisms, including diffusion, swelling, and erosion, can control the release of drugs from polymeric hydrogels. Among these mechanisms, diffusion-controlled release is the most common for releasing small drug molecules from a hydrogel, where the drug diffuses according to its concentration gradient. The small pore size of the hydrogel or the high molecular size of the drug limits diffusion. Swelling-controlled release can be achieved by drug desorption from the gel after water absorption into the gel’s intermolecular network. Meanwhile, the erosion mechanism refers to the drug release controlled by the kinetics of polymeric degradation [23,24,25].

Polymeric swelling combined with a slow degradation rate of the studied hydrogels is responsible for controlling drug release. The release of the drug is expected to be controlled by a combined diffusion and erosion mechanism, which allows for the regulation of drug release rates. The swollen hydrogel retains drug molecules within its intermolecular network for an extended period, slowly degrading to enable the release of the drug.

#### 2.2.4. Rheological Studies

The linear viscoelastic regions (LVRs) for all hydrogels were identified before assessing their frequency-dependent viscoelastic properties (elastic modulus (G′) and viscous modulus (G″) (Section 2.3). In this region, the hydrogels remained intact while oscillating over various frequencies to determine G′ and G″ as a function of frequency [26].

**Figure 4:**
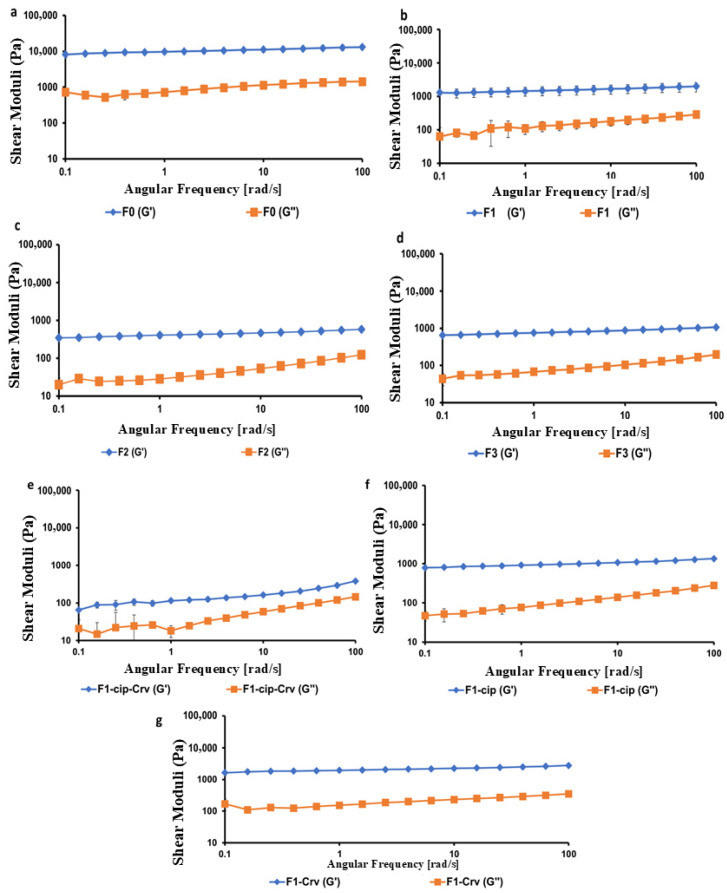
The frequency-dependence of elastic modulus (G′) and viscous modulus (G″) of (**a**) F0, (**b**) F1, (**c**) F2, (**d**) F3, (**e**) F1-cipr-Crv, (**f**) F1-cip, and (**g**) F1-Crv samples. Data are presented as mean ± SD (*n* = 3).

Hydrogels typically exhibit solid-like mechanical behavior, characterized by a higher storage modulus (G′) than the loss modulus (G″) across the tested frequency range. The rheological studies of hydrogels were performed at 25 °C, a temperature that provides a baseline for formulation development and allows for the assessment of hydrogels’ rheological properties at ambient temperature. Additionally, a temperature of 25 °C is used to assess the handling, dispensing, and injection of hydrogels [27,28]. Figure 4 illustrates the viscoelastic properties of all hydrogels at 25 °C, where G′ dominated G″, indicating a gel-like structure. The gel strength was determined by calculating the ratio of G′ to G″ at a frequency of 6.31 rad/s. This frequency is close to 6.28 rad/s (1 Hz), a value commonly used in oscillatory rheological studies for skin topical formulations and in defining the LVR of hydrogels [26].

Comparing the gel strengths (G′/G″ values) of all studied hydrogels reveals a decrease in the gel strength of F1 by 3.03 ± 0.16 compared to F2 and F3 hydrogels, which showed higher gel strengths of 9.79 ± 0.52 and 9.02 ± 0.51, respectively. The reduction in the F1 might be attributed to the lower HA: CS ratio of 1:1, compared to F2 and F3, which have higher ratios of 1:2 and 1:3, respectively, thereby enhancing the AH-CS physical interactions. These findings aligned with the viscoelastic properties of hydrogels, where F1-cip-Crv exhibited the lowest G′ and G″ values (Figure 4).

The gelation points (T_sol→gel_) and curing temperatures (T_-curing_) for all hydrogels, calculated from Anton Paar software during the temperature sweep studies, are illustrated in Table 1. The thermo-responsive in situ hydrogels should display a gelation temperature (_Tsol→gel_) above room temperature and convert to a gel at skin temperatures ranging between 32 and 35 °C [29,30]. Loaded hydrogels exhibited higher Tsol→gel than the non-loaded ones, which varies based on the hydrogels’ composition. Whereas T-curing refers to the temperature at which the hydrogel exceeds initial gelation (T_sol→gel_) and further strengthens and fully gels. Therefore, T-curing is always higher than T_sol→gel_ [31].

#### 2.2.5. In Vitro Release and Kinetics of Drug Release

**Figure 5:**
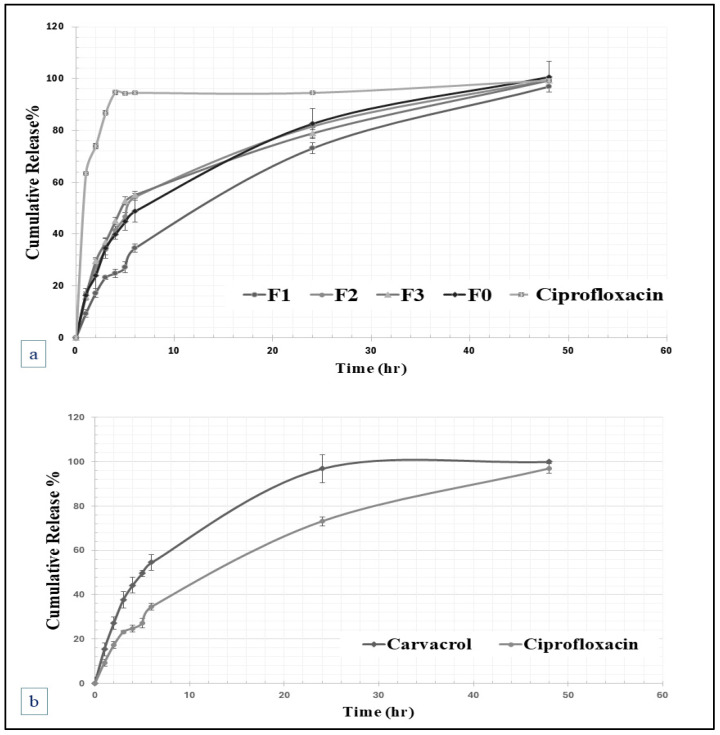
The in vitro release of (**a**) ciprofloxacin from F0-F3 hydrogels compared to that of raw ciprofloxacin, and for (**b**) Carvacrol and ciprofloxacin from F1-Cip-Crv hydrogel formulations (*n* = 3).

The release of ciprofloxacin from the F0-F3 hydrogels was evaluated and compared with that of the ciprofloxacin solution, as displayed in Figure 5. All the studied hydrogels sustained the drug release for more than 24 h, enhancing the effectiveness of the treatments. For instance, the cumulative amounts of ciprofloxacin released after 1 h were 63.34 ± 0.48% for ciprofloxacin solution, 16.38 ± 1.2% from F0 hydrogel, 9.33 ± 1.47% from F1 hydrogel, 19.8 ± 2.28% from F2 hydrogel, and 15.92 ± 0.96% from F3 hydrogel. Whereas the cumulative percentages of ciprofloxacin after three hours were 86.66 ± 5.72% for raw ciprofloxacin, 34.51 ± 3.96% from F0 hydrogel, 23.16 ± 0.75% from F1 hydrogel, 33.98 ± 2.07% from F2 hydrogel, and 36.94 ± 1.36% from F3 hydrogel.

The ability of these hydrogels to sustain the release of ciprofloxacin can be attributed to the interaction between the polymeric groups of hyaluronic acid and chitosan, as well as the electrostatic crosslinking with glycerophosphate. The swelling and degradation section further elucidates these slow drug release properties, as swelling combined with the slow degradation rates of the studied hydrogel can prolong the duration of drug release. The swollen hydrogel retains the drug within its network for an extended period while slowly degrading to enable its release.

According to the kinetic evaluation of the release rate, the release profiles were assessed kinetically through data fitting using Excel software with various mathematical models, including zero-order, first-order, Higuchi, Weibull, Hixon-Crowell, and Korsmeyer–Peppas models, which are commonly used to evaluate the release patterns of incorporated drug from polymeric matrices, and the release profiles were best fitted by the Higuchi release model, which is usually used to describe drug release from homogenous insoluble polymeric matrices, suggesting that the drug follows the Fickian diffusion process, where the square root of a time-dependent process fitted the release profile [32].

### 2.3. Antibacterial Activity

**Figure 6:**
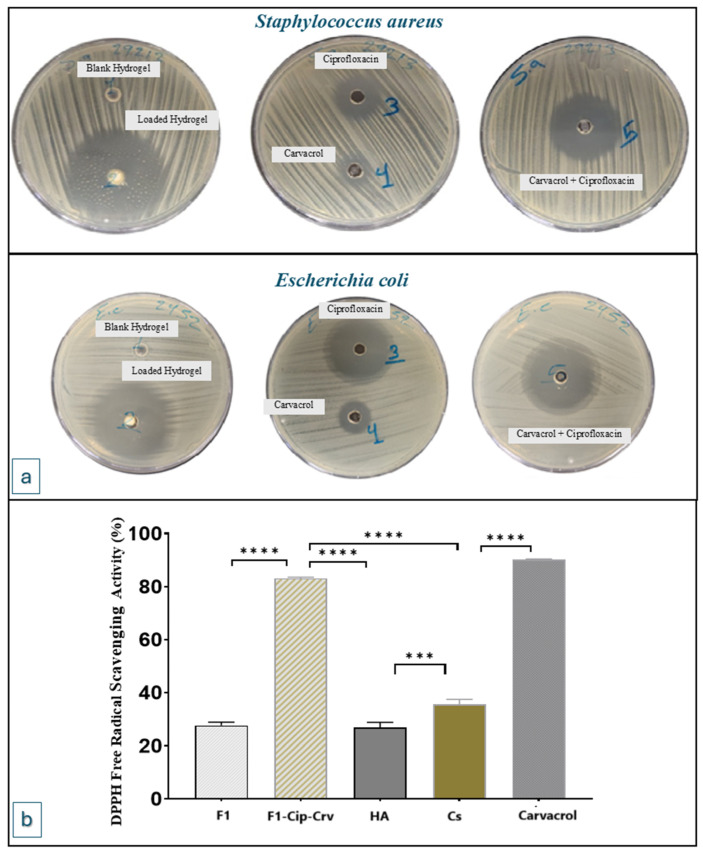
(**a**) Agar well diffusion test of compounds at 24 h against *Staphylococcus aureus* (ATCC 29213) and *Escherichia coli* (ATCC 2452) (*n* = 3). (**b**) DPPH free radical scavenging of F1 and F1-Cip-Crv (loaded hydrogel) and raw components at 25 °C (*n* = 8). *** *p* value < 0.001, **** *p* value < 0.0001.

The antibacterial effect of the (F1) hydrogels was assessed and compared with raw carvacrol and ciprofloxacin, and comparable mixtures of ciprofloxacin and carvacrol in the same ratios, tested against reference strains of *Staphylococcus aureus* (ATCC 29213) and *Escherichia coli* (ATCC 2452). The AWD study was used, and the results demonstrated varying antibacterial activity against the selected strains, as shown in Table 2 and Figure 6. Ciprofloxacin and carvacrol exhibit antibacterial activity, inhibiting the growth of bacteria. Ciprofloxacin is an efficient antibiotic with antibacterial activities against both studied bacterial strains. Carvacrol also has an antibacterial effect against both Gram-positive and Gram-negative bacterial strains [33].

While blank hydrogel did not demonstrate bacterial growth inhibition, the loaded hydrogel was more efficient than the corresponding ciprofloxacin and carvacrol mixture. This can be attributed to the antibacterial properties of chitosan, which are believed to work through various mechanisms. For instance, chitosan can form a polymer membrane that surrounds the bacterial cell wall, stopping bacterial metabolism by blocking the flow of essential nutrients and oxygen. Additionally, cationic chitosan can also penetrate the bacterial cell and disrupt the cytoplasmic membrane, resulting in cellular leakage and cell death [34].

Carvacrol exhibits antibacterial activities against both Gram-negative and Gram-positive bacteria, primarily by damaging the cell membrane, thereby increasing membrane permeability and disrupting the cell wall. Ciprofloxacin also has an antibacterial effect against both Gram-positive and Gram-negative bacterial strains. The synergistic antibacterial activity of combined ciprofloxacin and carvacrol has been reported for several bacterial strains [33,35,36]. The study results demonstrated the synergistic antibacterial activity of carvacrol and ciprofloxacin on both bacterial strains of *Staphylococcus aureus* and *Escherichia coli* (Section 4.2).

### 2.4. Antioxidant DPPH Solution Scavenging Activity

The antioxidant properties of the blank gel (F1) and carvacrol-ciprofloxacin-loaded hydrogel (F1-Cip-Crv) and raw components were assessed using the DPPH radical test. This test relies on the H-donating antioxidant reducing DPPH to a yellow-colored molecule [37]. Antioxidant activities are illustrated in Figure 6b, where the results indicate that the antioxidant activity of the loaded hydrogel is primarily attributed to the incorporation of carvacrol.

The antioxidant effect of carvacrol has been previously approved via multiple in vivo and in vitro investigations. Its ability to hinder oxidation is primarily attributed to the existence of a hydroxyl group in its aromatic structure [38]. Research has demonstrated that carvacrol eliminates free radicals and reactive oxygen species [39,40].

While the antioxidant activity of the developed gel was evaluated using the DPPH scavenging assay, future investigations could include additional oxidation activity testing to confirm and expand the assessment of the antioxidant potential of the developed system.

### 2.5. Animal Wound Healing Studies

**Figure 7:**
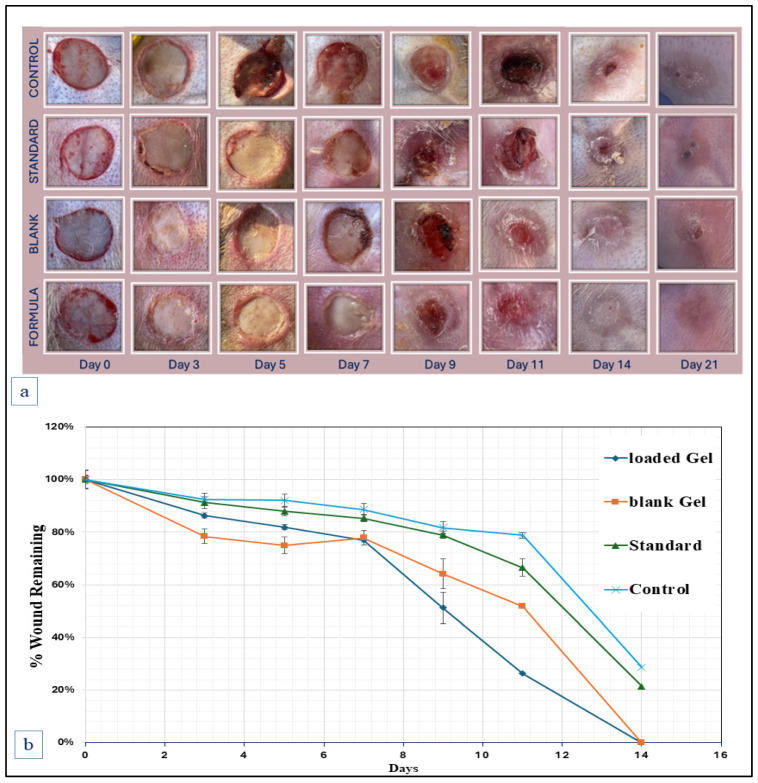
Wound healing study results on the rabbit model. (**a**) Photographic illustration of wound progression for control, standard, blank hydrogel (F1), and loaded hydrogel (F1-Cip-Carv) from 0 to 14 days of treatment in experimental groups. (**b**) Quantitative representation of wound healing (%) over 14 days in the experimental groups.

In vivo, wound healing studies were conducted to evaluate blank F1 hydrogel and drug-loaded F1-Cip-Crv hydrogels. Figure 7 presents a visual overview of the wound-healing process. Wound closures were observed for control, standard, blank, and loaded hydrogels. The wound areas of all groups gradually narrowed throughout the study, and both blank and loaded F1 hydrogel groups showed accelerated wound closure compared to the control groups. As presented in the quantitative wound area percentages (Figure 7), on day 3, the mean wound areas were 92.4%, 91.2%, 78.4%, and 86.3%. And on Day 9, the percentages were 81.8%, 79.0%, 64.2%, and 51.2% for the negative control, standard, blank gel (F1), and loaded gel (F1-Cip-Crv), respectively. A significant difference was apparent between the blank and loaded hydrogel groups and the negative control and standard groups. After 14 days, the wounds in all hydrogel groups had closed entirely. Meanwhile, the wound area percentages for the control and standard groups were 28.7% and 21.5%, respectively.

The results showed that a blank hydrogel can accelerate wound healing, which can be attributed to the hydration and moisture absorption effects of the hydrogel, as well as the healing ability and intrinsic properties of hyaluronic acid and chitosan. The further acceleration in wound closure for the loaded hydrogel is related to the antibacterial and antioxidant properties of ciprofloxacin and carvacrol.

The positive control group (standard) was employed to evaluate the developed hydrogel formulation in comparison to the commonly marketed topical product, povidone-iodine. The wound-treated groups with both blank and drug-loaded hydrogels demonstrated faster healing rates compared to the standard group, indicating the superior wound-healing ability of the developed thermoresponsive gel compared with commonly used marketed products.

The proposed hydrogel exhibits excellent features for multifunctional wound healing treatments, including good antibacterial activity, thermo-responsive in situ gelation at skin temperature, swelling capabilities, and prolonged drug release properties. In response to these findings, in vivo, wound healing trials were conducted in rabbits. This decision was made because rabbits’ skin is similar to human skin in terms of aging and healing time, rendering it suitable as a model for potential treatment methods [41,42]. In this model, a full-thickness excisional wound was created with an avascular base and low wound contraction. In addition, the rabbit ear’s ventral surface is flat and thin, making it perfect for uniform wound production, observation, and measuring [43,44].

Assessing the toxicity and irritation potential of hydrogel formulations is essential for their safe and effective application and should be addressed in future studies. In our animal experiments, no skin irritation or other adverse effects were observed in any of the experimental groups. Previous studies have reported that hyaluronic acid and its salts are considered safe for cosmetic use [45]. Furthermore, hemolysis, acute toxicity, and cytotoxicity tests have demonstrated that chitosan and glycerophosphate exhibit good biocompatibility [46].

### 2.6. Histopathologic Examination (H&E Stain)

**Figure 8:**
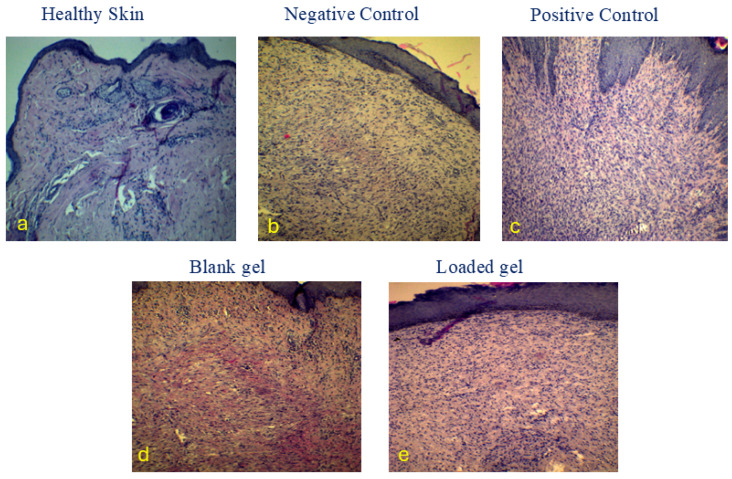
Photomicrographs of HE-stained histological sections represent the healing wound area in different rabbits after 21 days of the experiment. (**a**) Healthy Skin, (**b**) Negative control: Treated with normal saline, (**c**) Positive control: Treated with iodine, (**d**) Blank gel (F1), and (**e**) F1-cip-Crv gel.

Photomicrographs of HE-stained histological sections represent the healing wound area after 21 days of the experiment. Figure 8a. Healthy skin (4×) shows an intact epidermis. In the dermis, hair follicles and adnexal glands appear fully developed with densely packed collagen. The negative control (10×) photomicrograph shows complete reepithelialization with minimal keratinization, prominent granulation tissue with a high cellular content, neo-vascularization, and numerous fibroblasts (Figure 8b). The collagen fibers appear loosely organized and have not yet been densely compacted, indicating an early stage of remodeling.

Additionally, inflammatory cells infiltrated significantly, most likely lymphocytes and macrophages. The positive control (10×) shows complete active reepithelialization with a thick epidermis and mature granulation tissue, exhibiting greater collagen deposition. Collagen fibers are more densely packed and better organized (Figure 8c), and mild inflammatory infiltration is observed, reflecting the resolution of the acute phase. Neo-vascularization is less prominent. The blank gel (10×) shows complete active reepithelialization with a thick epidermis. The underlying dermis contains granulation tissue that has begun to mature (Figure 8d). Collagen fibers are present with ongoing synthesis, and remodeling is in progress, as indicated by the presence of plentiful fibroblasts and moderate cellularity. There is mild to moderate inflammatory cell infiltration, consistent with the subacute stage of healing. The loaded hydrogel (10×) group exhibits the most advanced healing among all wound group samples (Figure 8e). Epithelialization is effectively complete, with a well-formed epidermis that is markedly thick and nearly approaches the architecture of normal skin. Mature granulation tissue that is rich in collagen is observed, and collagen deposition is relatively well-organized, showing signs of remodeling (with collagen bundles aligning parallel to the epidermal surface), and minimal inflammatory cell infiltration is seen. All these changes are hallmarks of an advanced healing process; epithelialization was most pronounced and fastest with the hydrogel, followed by the standard and, to a lesser extent, the negative control.

By day 21, F1-cip-Crv hydrogel showed the best healing results, producing histological features similar to those of normal skin. It excelled in saline therapy by accelerating closure and collagen deposition. The blank gel confirmed that a moist gel dressing provides healing advantages over saline, even in the absence of an active component. The findings demonstrate that both blank and loaded hydrogels, used as wound-healing treatments, have notable regenerative advantages, enhancing connective tissue repair and epithelial regeneration while reducing chronic inflammation. This suggests that the GP-loaded gel is a promising therapeutic approach for improving wound healing, leading to stronger and more mature tissue repair. The hydrogel facilitates collagen regeneration by functioning as a porous, hydrated scaffold that supports fibroblast adhesion, migration, and proliferation while preserving an ideal moist environment. Furthermore, hyaluronic acid and chitosan work in conjunction to activate TGF-β and VEGF signaling pathways, which in turn trigger fibroblasts to produce and arrange collagen fibers. This results in collagen bundles that are histologically denser, mature, and better aligned during tissue remodeling [47].

## 3. Conclusions

The developed hyaluronic–chitosan–glycerophosphate in situ-forming hydrogel combines rapid thermo-responsive gelation at 33 °C and has the flexibility to spread on irregular wound geometries. Incorporating ciprofloxacin and carvacrol into the formula maintained the mechanical strength of the formula, with a G′/G″ ≈ ratio of approximately 15.6, interconnected porosity, and controlled swelling. It enhanced antimicrobial activity against both *S. aureus* and *E. coli*. On the other hand, the formula exhibited sustained release following the Higuchi diffusion mechanism. In vivo, an ischemic rabbit ear model evaluation demonstrated accelerated wound closure on day 9, with a 51.2% residual area compared to 92.4% in the control group. Histological evidence showed advanced re-epithelialization, collagen maturation, and reduced inflammation. The results indicate the suitability and potential of the developed drug delivery system as multifunctional wound healing formulations.

## 4. Materials and Methods

### 4.1. Materials

Hyaluronic acid (HA, high Molecular weight of 1200 kDa) was purchased from China Xi’an Natural Biotechnology Company (Xi’an, China). Chitosan (Cs), with a low molecular weight (50–190 kDa, 75% deacetylated), was obtained from BIOSYNTH (YC158300, Lot: 0000029470, Staad, Switzerland). Sodium Glycerophosphate (GP) and carvacrol (5-isopropyl-2-methyl phenol) were supplied from GENOCHEM World (Valencia, Spain). Ciprofloxacin hydrochloride monohydrate was obtained from Tokyo Chemical Industry TCI (Tokyo, Japan). Potassium dihydrogen phosphate (KH_2_PO_4_) was from AZ Chem for chemicals (Thunder Bay, ON, Canada), and dipotassium hydrogen phosphate (K_2_HPO_4_) was from Xilong (Shantou, China). All other chemicals and reagents were of HPLC or analytical grade.

### 4.2. Methods

#### Gel Preparation

A 3% *w*/*v* chitosan (CS) was dissolved in 0.1 M acetic acid solution under magnetic stirring at 350 rpm for 4 h. Hyaluronic acid-glycerol phosphate (HA-GP) solutions were prepared by dissolving 60% *w*/*v* glycerol phosphate (GP) and 3% *w*/*v* hyaluronic Acid (HA) in deionized water under a magnetic stirrer at 350 rpm. These solutions were kept in the refrigerator for further use. HA-GP solution was added to the CS solution in an ice bath at a fixed addition rate of 0.5 mL/min using a syringe pump, with continuous stirring at 350 rpm. Loading the ciprofloxacin was performed in the CS solution. Carvacrol was incorporated into the gel using ultrasonication. Carvacrol was added to the prepared mixture in the final step of mixing with continuous sonication for 5 min to form a homogeneous mixture.

Table 1 displays the compositions of CS/HA/GP hydrogels. All gels kept the total polymeric concentration constant (3%). Blank hydrogels (F0-F3) were prepared using the same process without adding active ingredients (ciprofloxacin and carvacrol), and F0 consisted of CS mixed with a GP aqueous solution without HA. Table 3 summarizes the different prepared formulas.

**Figure 9:**
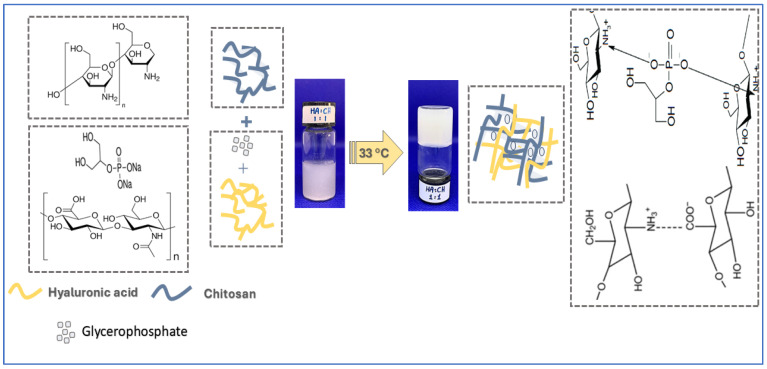
Schematic representation of CS/HA/GP hydrogel preparation.

To test thermoresponsive in situ gel formation, the mixture was placed in a warm water thermostatic bath at 33 °C, which mimics body skin temperature [48,49]. The formation of gel was observed with time (Figure 9).

### 4.3. Physicochemical Characterization:

#### 4.3.1. Fourier Transform Infrared Spectroscopy (FTIR) Analysis

The chemical structures of the produced hydrogel components were verified using FTIR spectroscopy. For raw materials, loaded, and drug-free blank hydrogels, a spectrum was recorded in the range of 4000–400 cm^−1^.

#### 4.3.2. Scanning Electron Microscopy SEM Analysis

SEM analysis was performed on the produced hydrogel using a Jeol JSM-5300 (Japan Electron Optics Laboratory, Tokyo, Japan). After placing the lyophilized hydrogel on stubs, a Q150R Rotary Pumped Sputter (Quorum Technologies, London, UK) was used to vacuum-coat it with platinum.

#### 4.3.3. Swelling and In Vitro Degradation Studies

To calculate the swelling and degradation properties of the prepared hydrogels, an accurately weighed hydrogel was soaked in PBS buffer at 37 °C [50]. The buffer was removed at predetermined intervals, and the hydrogel surface was gently wiped using filter paper. Then, the weight changes were recorded. The weight change percentage of the hydrogel was calculated using the following equation [51,52]:

(1) $$\mathrm{SR}\%\;=\frac{Wt-Wi}{Wi}\times 100$$

where (*Wi*) is the initial hydrogel weight and (*Wt*) is the swollen hydrogel weight at each time interval.

To quantify the prepared degradation ratios, the initial weight of *Wt*1 and the weight of the hydrogel, *Wt*2, were recorded at predetermined intervals. The degradation ratios were calculated using the following equation [51]:

(2) $$DR\%\frac{Wt1-Wt2}{Wt1}\times 100$$

### 4.4. Rheological Study

The rheological properties of the hydrogels were evaluated using a controlled-stress rheometer (CSR) (Anton Paar, MCR 302; Graz, Austria) with a cone-plate system of 25 mm diameter and a 1° cone angle, as described previously [53]. To determine the linear viscoelastic region (LVR) of the samples, a strain sweep test was conducted at 25 °C and at a constant frequency of 6.28 rad/s. A 0.5 g sample of the in situ gel was placed on the plate, and the cone was lowered to reach a trim gap of 0.5 mm. 

To evaluate the viscoelastic properties and gel strength of the in situ gels, a frequency sweep test was conducted at 25 °C, over a frequency range of 0.1–100 rad/s, with a constant strain of 0.1% chosen within the LVR based on the strain-sweep test. The ratio of storage modulus (G′) to loss modulus (G″) was determined to evaluate the strength of each in situ gel. The higher the G′/G″ ratio, the higher the strength of the gel network [54].

A temperature sweep test was conducted to determine the gelation temperature (T_sol→gel_) and curing temperature (T_-curing_) of the hydrogels. Samples were heated from 20 to 50 °C at a heating rate of 2 °C/min and a constant shear rate of 50 s^−1^. The initial temperature at which the in situ hydrogel converted from a sol state to a gel state (T_sol→gel_) and then the hydrogels were fully gelled (T_-curing_) were recorded. 

### 4.5. Drug Content

To calculate the drug content, 10 mg of hydrogel was dissolved in 10 mL of phosphate-buffered saline (PBS) and 0.1 M acetic acid at a 1:1 volume ratio. Then, the mixture was sonicated until the gel was dissolved completely. After that, the sample was analyzed using a UV-Vis spectrometer (C-7200, PEAK INSTRUMENTS, Houston, TX, USA). 

### 4.6. In Vitro Release Test

The in vitro release study was performed using the diffusion method. 1 mL of hydrogel was added to 7 cm dialysis tubes with a 12–14 kDa cutoff—the release medium consisted of 40 mL of PBS (pH 7.4) buffer. The bottle was incubated in a warm water bath at 33 °C. At predetermined time intervals, an aliquot was withdrawn from the release medium, refilled with fresh release medium, and analyzed using a UV-Vis spectrometer.

The release profiles of ciprofloxacin were assessed kinetically through data fitting using Excel software, 2016 with various mathematical models, including zero-order, first-order, Higuchi, Weibull, Hixon-Crowell, and Korsmeyer–Peppas models, which are commonly used to evaluate the release patterns of active ingredients from polymeric matrices [55,56].

### 4.7. Antibacterial Activity Test

Agar well diffusion was carried out as described by Jaradat et al. [57], with some changes. Bacterial strains *Staphylococcus aureus* ATCC 29213 and *Escherichia coli* ATCC 2452 were cultivated on a plate of Tryptone Soy agar (Oxoid, UK) at 37 °C overnight. After that, one to three colonies were suspended in one milliliter of sterile saline with a 0.9% concentration and adjusted to 0.5 McFarland at 625 nm. After being poured onto sterile Petri dishes, 25 mL of sterile Mueller-Hinton agar (Oxoid, UK) was left to solidify. To achieve a consistent bacterial density on the agar surface, sterile cotton swabs dipped in bacterial suspensions were wiped over the whole plate surface when inoculating plates with the test strains. A sterile glass cork borer cut wells (6 mm) into each agar plate. Wells were filled with 50 μL of (1—Blank Hydrogel, 2—Loaded Hydrogel, 3—Ciprofloxacin, 4—Carvacrol, and 5—Ciprofloxacin–Carvacrol).

Plates were placed in a laminar flow cabinet at ambient temperature for 15 min to allow diffusion before being incubated aerobically at 37 °C for 24 and 48 h. The diameter of the inhibitory zones was measured to evaluate the antibacterial activity. The experiment was repeated in triplicate.

### 4.8. Antioxidant DPPH Solution Scavenging Test

Preparation of 0.1 mM of DPPH in Ethanol. This solution was kept at room temperature and covered with aluminum foil for 2 h to stabilize. Then, 2 mL of freshly prepared hydrogel samples were added to 10 mL of DPPH solution. This mixture was stirred for 20 min to ensure adequate mixing, and then it was incubated at 25 °C for 25 min. A 2 mL control sample of ethanol was dissolved in 10 mL of DPPH solution. 150 mL of each formulation was placed on a well plate (with 8 replicates for each sample), and the absorbance was measured at a wavelength of 517 nm using a microplate reader.

### 4.9. In Vivo Wound Healing Study

According to previous reports [58]. The rabbit ear hypertrophic ulcer model was used. The Ethics Committee at Yarmouk University approved the animal trial protocols (IACUC/2025/5).

Five New Zealand White female rabbits (2–2.5 kg) were used and anesthetized using IM injection of 60 mg/kg ketamine and 5 mg/kg xylazine. Each rabbit received four wounds on the ventral side of the ear, with each wound assigned to a different treatment condition. Negative Control: wounds left untreated; Positive Control: wounds treated with commercially available treatment; Blank: wounds treated with blank (non-drug containing hydrogel); loaded Gel (F1-cip-Crv): wounds treated with active ingredients loaded hydrogel. Ischemia was induced in the rabbits’ ears by ligating the central and rostral arteries. To ensure controlled ischemic wound conditions and reproducibility, visual observation of tissue blanching was used to validate the status of ischemic wounds in all animal groups [58]. Wounds were created using a 10 mm dermal biopsy punch on the ventral side of the ears. Using a dissecting microscope, the epidermis, dermis, and perichondrium were removed without dissection of the cartilage. Hydrogel was applied to cover the wounds (100 µL), and the area was observed for 3 min to ensure gel formation. The wound was then covered with Tegaderm film, and wound healing was evaluated for 21 days. On day 21, skin samples were resected, fixed in formalin, and embedded in paraffin for histological examination.

The control group was rinsed with normal saline without treatment, and the standard therapy of marketed povidone, blank, and (F1-cip-Crv) drug-loaded hydrogels was evaluated in groups 3 and 4, respectively.

Wound area measurement was performed using digital photographs combined with a digital caliper. To ensure consistent measurements, a standardized camera setup with high resolution was used to capture images. The images were then analyzed using ImageJ software (ImageJ, 1.x 1.54p), to calculate the wound area. A scale bar was used for calibration to ensure accurate measurements. All measurements were performed by one trained operator, with blinding of animal groups to ensure consistency and prevent bias in measurements.

The remaining wound area percentages were calculated by dividing the wound area at each time point by the original (day 0) wound area to allow comparison of wound healing progress. All measurements were repeated in triplicate.

### 4.10. Histopathologic Examination (H&E Stain)

The collected tissue samples were immersed in 10% buffered formalin for 48 h. Specimens were subsequently processed through a graded series of alcohols and xylene, removing water and thoroughly clearing the tissue for paraffin embedding afterward. Tissue processing involves several stages, including dehydration, clearing, impregnation, and embedding, each of which lasts a specific duration, ultimately ensuring the completion of the procedure.

After fixation and processing, the tissues were embedded in paraffin blocks. Using a microtome, sections 3–5 µm thick were cut from the paraffin blocks. Following xylene deparaffinization, the sections were rehydrated using a graded alcohol series and then stained with hematoxylin. Differentiation was done with 0.3% acid alcohol, and then sections were alkalinized somewhat afterward, enhancing nuclear contrast. Eosin counterstain was subsequently applied to highlight cytoplasmic and extracellular components. The specimen was then thoroughly dehydrated and cleared in xylene. Afterward, it was mounted using DPX. The stained slides were examined under a light microscope at low (×10), medium (×40), and high magnification (×100). Nuclear morphology, cytoplasmic characteristics, and extracellular matrix features were assessed to evaluate histopathologic changes.

### 4.11. Statistical Analysis

The mean value and standard deviation were presented, and each sample was repeated three times. A one-way ANOVA was performed, followed by Tukey’s multiple comparisons using GraphPad Prism.9, *p*-values of less than 0.05 were considered statistically significant.

## Figures and Tables

**Table 1:** The results of the rheological studies of hydrogels.

Sample	LVR (%)	Gel Strength (G′/G″) at 6.31 Rad/s	Gelation Point (T_sol→gel_, °C)	Curing Temperature (T_-curing_, °C)
F0	0.01–15.8	10.24 ± 0.40	25.11 ± 0.76	30.55 ± 1.80
F1	0.01–6.31	9.73 ± 0.50	26.00 ± 0.49	34.14 ± 3.48
F2	0.01–25.1	9.79 ± 0.52	31.49 ± 0.63	38.30 ± 0.42
F3	0.01–2.51	9.02 ± 0.51	31.97 ± 0.82	38.19 ± 1.40
F1-cip-Crv	0.01–10.0	3.03 ± 0.16	28.94 ± 0.36	35.91 ± 1.50
F1-cip	0.01–15.8	8.34 ± 0.39	28.91 ± 0.66	37.35 ± 0.82
F1-Crv	0.01–25.1	10.00 ± 0.72	32.14 ± 1.20	38.52 ± 5.30

**Table 2:** Antibacterial activity of compounds evaluated by agar well diffusion method at 24 h *.

Sample	Zone of Inhibition (mm)	
	*Staphylococcus aureus* ATCC 29213	*Escherichia coli* ATCC 2452
F1-Cip-Crv	40 ± 0.01	40 ± 0.01
Ciprofloxacin	18 ± 1.15	28.7 ± 0.57
Carvacrol	13 ± 0.01	15.7 ± 0.57
Ciprofloxacin-Carvacrol	30 ± 0.01	34.7 ± 0.57

* Diameter of inhibition zone (mm) presented as means ± SE.

**Table 3:** Compositions, pH, and thermoresponsive in situ gel formation time at 33 °C of the evaluated CS/HA/GP hydrogels (*n* = 3), all hydrogels kept the total polymeric concentration constant (3%).

Hydrogel	Polymeric Ratio (HA:CS)	GP (%*w*/*v*)	Ciprofloxacin (mg/mL)	Carvacrol (µL)	pH	Gelation Time at 33 °C (min)
F0	(0:1)	60	14	600	8.01	3.2 ± 0.3
F1	(1:1)	60	-	-	7.83	3.2 ± 0.3
F2	(1:2)	60	14	600	7.7	3.2 ± 0.3
F3	(1:3)	60	14	600	7.46	3.7 ± 0.8
F1-Cip-Crv	(1:1)	60	14	600	7.63	3.8 ± 0.3
F1-Cip	(1:1)	60	14	-	7.77	3.2 ± 0.3
F1-Crv	(1:1)	60	-	600	7.83	3.7 ± 0.3
PM	(1:1)	-	-	-	-	-

## Data Availability

The datasets analyzed in the current study are available from the corresponding author, HM, upon request.

## Supplementary Materials

The following supporting information can be downloaded at: https://www.mdpi.com/article/10.3390/gels11100835/s1, Table S1: FTIR Assignment of raw Hyaluronic acid, Chitosan and Ciprofloxacin.

## References

[1] Başhan V., Yucesan M., Demirel H., Gul M. (2022). Health, safety, and environmental failure evaluation by hybridizing fuzzy multi-attribute decision-making methods for maritime scrubber systems. Environ. Monit. Assess..

[2] Burdick J.A., Prestwich G.D. (2011). Hyaluronic acid hydrogels for biomedical applications. Adv. Mater..

[3] PubChem Hyaluronic Acid Sodium Bethesda, MD: National Center for Biotechnology Information. https://pubchem.ncbi.nlm.nih.gov/compound/Hyaluronic-acid-sodium.

[4] Gallo N., Nasser H., Salvatore L., Natali M.L., Campa L., Mahmoud M., Capobianco L., Sannino A., Madaghiele M. (2019). Hyaluronic acid for advanced therapies: Promises and challenges. Eur. Polym. J..

[5] Mashaqbeh H., Obaidat R., Rezigue M., Omari D., Shakhatreh G. (2024). Ferric ions crosslinked hyaluronic acid beads: Potentials for drug delivery use. Drug Dev. Ind. Pharm..

[6] Dragan E.S. (2014). Design and applications of interpenetrating polymer network hydrogels. A review. Chem. Eng. J..

[7] Mashaqbeh H., Hamed R., Obaidat R., Hmedat A., Aburayya R., Hijazi S., Akkam Y. (2025). Hyaluronic acid and K-carrageenan metal ionic cross-linked polymers: A promising injectable hydrogels for prolonged chemotherapeutic drug delivery. J. Biomater. Sci. Polym. Ed..

[8] Kou S.G., Peters L.M., Mucalo M.R. (2021). Chitosan: A review of sources and preparation methods. Int. J. Biol. Macromol..

[9] Chenite A., Buschmann M., Wang D., Chaput C., Kandani N. (2001). Rheological characterisation of thermogelling chitosan/glycerol-phosphate solutions. Carbohydr. Polym..

[10] Aranaz I., Alcántara A.R., Civera M.C., Arias C., Elorza B., Heras Caballero A., Acosta N. (2021). Chitosan: An overview of its properties and applications. Polymers.

[11] Zhou H.Y., Jiang L.J., Cao P.P., Li J.B., Chen X.G. (2015). Glycerophosphate-based chitosan thermosensitive hydrogels and their biomedical applications. Carbohydr. Polym..

[12] Berger J., Reist M., Mayer J., Felt O., Peppas N., Gurny R. (2004). Structure and interactions in covalently and ionically crosslinked chitosan hydrogels for biomedical applications. Eur. J. Pharm. Biopharm..

[13] Peers S., Montembault A., Ladavière C. (2020). Chitosan hydrogels for sustained drug delivery. J. Control. Release.

[14] Supper S., Anton N., Seidel N., Riemenschnitter M., Curdy C., Vandamme T. (2014). Thermosensitive chitosan/glycerophosphate-based hydrogel and its derivatives in pharmaceutical and biomedical applications. Expert Opin. Drug Deliv..

[15] Chenite A., Chaput C., Wang D., Combes C., Buschmann M.D., Hoemann C., Leroux J., Atkinson B., Binette F., Selmani A. (2000). Novel injectable neutral solutions of chitosan form biodegradable gels in situ. Biomaterials.

[16] Chenite A., Chaput C., Combes C., Selmani A., Jalal F. (2002). Temperature-Controlled pH-Dependent Formation of Ionic Polysaccharide Gels. Google Patents.

[17] Zhang W., Jin X., Li H., Zhang R.-R., Wu C.-W. (2018). Injectable and body temperature sensitive hydrogels based on chitosan and hyaluronic acid for pH sensitive drug release. Carbohydr. Polym..

[18] Weng H., Jia W., Li M., Chen Z. (2022). New injectable chitosan-hyaluronic acid based hydrogels for hemostasis and wound healing. Carbohydr. Polym..

[19] Liu S., Jiang N., Chi Y., Peng Q., Dai G., Qian L., Xu K., Zhong W., Yue W. (2022). Injectable and self-healing hydrogel based on chitosan-tannic acid and oxidized hyaluronic acid for wound healing. ACS Biomater. Sci. Eng..

[20] Li J., Su J., Liang J., Zhang K., Xie M., Cai B., Li J. (2024). A hyaluronic acid/chitosan composite functionalized hydrogel based on enzyme-catalyzed and Schiff base reaction for promoting wound healing. Int. J. Biol. Macromol..

[21] Rahmanian-Devin P., Baradaran Rahimi V., Askari V.R. (2021). Thermosensitive chitosan--β--glycerophosphate hydrogels as targeted drug delivery systems: An overview on preparation and their applications. Adv. Pharmacol. Pharm. Sci..

[22] El-Aassar M., El Fawal G., Kamoun E.A., Fouda M.M. (2015). Controlled drug release from cross-linked κ-carrageenan/hyaluronic acid membranes. Int. J. Biol. Macromol..

[23] Rizwan M., Yahya R., Hassan A., Yar M., Azzahari A.D., Selvanathan V., Sonsudin F., Abouloula C.N. (2017). pH sensitive hydrogels in drug delivery: Brief history, properties, swelling, and release mechanism, material selection and applications. Polymers.

[24] Rao K.R., Devi K.P. (1988). Swelling controlled-release systems: Recent developments and applications. Int. J. Pharm..

[25] Lin C.-C., Metters A.T. (2006). Hydrogels in controlled release formulations: Network design and mathematical modeling. Adv. Drug Deliv. Rev..

[26] Hamed R., Abu Alata W., Abu-Sini M., Abulebdah D.H., Hammad A.M., Aburayya R. (2023). Development and Comparative Evaluation of Ciprofloxacin Nanoemulsion-Loaded Bigels Prepared Using Different Ratios of Oleogel to Hydrogels. Gels.

[27] Gaia Z., Barbara V., Caterina V., Marco R., Giuseppina S., Silvia R. (2025). Exploring Sangelose-cyclodextrin in situ-forming hydrogels for vitreous substitution: Design, development, and characterization. Mater. Des..

[28] Chen M.H., Wang L.L., Chung J.J., Kim Y.-H., Atluri P., Burdick J.A. (2017). Methods to assess shear-thinning hydrogels for application as injectable biomaterials. ACS Biomater. Sci. Eng..

[29] Okur M.E., Ayla Ş., Batur Ş., Yoltaş A., Genç E., Pertek S., Okur N.Ü. (2019). Evaluation of In Situ Gel Containing Pycnogenol for Cutaneous Wound Healing. Medeni. Med. J..

[30] Lee C.M., Jin S.-P., Doh E.J., Lee D.H., Chung J.H. (2019). Regional variation of human skin surface temperature. Ann. Dermatol..

[31] Fairuz A., Sapuan S., Zainudin E., Jaafar C. (2015). The effect of gelation and curing temperatures on mechanical properties of pultruded kenaf fibre reinforced vinyl ester composites. Fibers Polym..

[32] Paul D. (2011). Elaborations on the Higuchi model for drug delivery. Int. J. Pharm..

[33] Shemchuk O., d’Agostino S., Fiore C., Sambri V., Zannoli S., Grepioni F., Braga D. (2020). Natural antimicrobials meet a synthetic antibiotic: Carvacrol/thymol and ciprofloxacin cocrystals as a promising solid-state route to activity enhancement. Cryst. Growth Des..

[34] Chen X., Liu H., Yang Y., Li P., Wang X., Zhang K., Zeng K., Ming J., Lei X. (2025). Chitosan-based emulsion gel beads developed on the multiple-unit floating delivery system for gastric sustained release of proanthocyanidins. Food Hydrocoll..

[35] Gan C., Langa E., Wang G., Van Bambeke F., Ballestero D., Pino-Otín M.R. (2025). Mechanisms of action and resistance prevention of synergistic thymol and carvacrol combinations with antibiotics in Staphylococcus aureus and Acinetobacter baumannii. Nat. Prod. Bioprospecting.

[36] Akhlaq A., Ashraf M., Omer M.O., Altaf I. (2023). Synergistic antibacterial activity of carvacrol loaded chitosan nanoparticles with Topoisomerase inhibitors and genotoxicity evaluation. Saudi J. Biol. Sci..

[37] Li J., Shi X., Yang K., Guo L., Yang J., Lan Z., Guo Y., Xiao L., Wang X. (2024). Fabrication and characterization of carvacrol encapsulated gelatin/chitosan composite nanofiber membrane as active packaging material. Int. J. Biol. Macromol..

[38] Aristatile B., Al-Numair K.S., Al-Assaf A.H., Veeramani C., Pugalendi K.V. (2015). Protective effect of carvacrol on oxidative stress and cellular DNA damage induced by UVB irradiation in human peripheral lymphocytes. J. Biochem. Mol. Toxicol..

[39] Samarghandian S., Farkhondeh T., Samini F., Borji A. (2016). Protective effects of carvacrol against oxidative stress induced by chronic stress in rat’s brain, liver, and kidney. Biochem. Res. Int..

[40] Potra Cicalău G.I., Ciavoi G., Scrobotă I., Marcu A.O., Romanul I., Marian E., Vicaș L.G., Ganea M. (2023). Assessing the Antioxidant Benefits of Topical Carvacrol and Magnolol Periodontal Hydrogel Therapy in Periodontitis Associated with Diabetes in Wistar Rats. Dent. J..

[41] Sami D.G., Heiba H.H., Abdellatif A. (2019). Wound healing models: A systematic review of animal and non-animal models. Wound Med..

[42] Masson-Meyers D.S., Andrade T.A., Caetano G.F., Guimaraes F.R., Leite M.N., Leite S.N., Frade M.A.C. (2020). Experimental models and methods for cutaneous wound healing assessment. Int. J. Exp. Pathol..

[43] Breen A., Mc Redmond G., Dockery P., O’Brien T., Pandit A. (2008). Assessment of wound healing in the alloxan-induced diabetic rabbit ear model. J. Investig. Surg..

[44] O’Loughlin A., Kulkarni M., Vaughan E.E., Creane M., Liew A., Dockery P., Pandit A., O’bRien T. (2013). Autologous circulating angiogenic cells treated with osteopontin and delivered via a collagen scaffold enhance wound healing in the alloxan-induced diabetic rabbit ear ulcer model. Stem Cell Res. Ther..

[45] Becker L.C., Bergfeld W.F., Belsito D.V., Hill R.A., Klaassen C.D., Liebler D.C., Marks J.G., Shank R.C., Slaga T.J., Snyder P.W. (2011). Final report of the cosmetic ingredient review expert panel safety assessment of polymethyl methacrylate (PMMA), methyl methacrylate crosspolymer, and methyl methacrylate/glycol dimethacrylate crosspolymer. Int. J. Toxicol..

[46] Hoemann C.D., Chenite A., Sun J., Hurtig M., Serreqi A., Lu Z., Rossomacha E., Buschmann M.D. (2007). Cytocompatible gel formation of chitosan-glycerol phosphate solutions supplemented with hydroxyl ethyl cellulose is due to the presence of glyoxal. J. Biomed. Mater. Res. Part A.

[47] Deng Y., Ren J., Chen G., Li G., Wu X., Wang G., Gu G., Li J. (2017). Injectable in situ cross-linking chitosan-hyaluronic acid based hydrogels for abdominal tissue regeneration. Sci. Rep..

[48] Liu Y., Wang L., Liu J., Di Y. (2013). A study of human skin and surface temperatures in stable and unstable thermal environments. J. Therm. Biol..

[49] Caldwell J.N., Matsuda-Nakamura M., Taylor N.A. (2014). Three-dimensional interactions of mean body and local skin temperatures in the control of hand and foot blood flows. Eur. J. Appl. Physiol..

[50] Tarawneh O., Abu Mahfouz H., Hamadneh L., Deeb A.A., Al-Sheikh I., Alwahsh W., Abed A.F. (2022). Assessment of persistent antimicrobial and anti-biofilm activity of p-HEMA hydrogel loaded with rifampicin and cefixime. Sci. Rep..

[51] Wang W., Shi D., Zhang Y., Li W., Li F., Feng H., Ma L., Yang C., Peng Z., Song G. (2023). An injectable hydrogel based on hyaluronic acid prepared by Schiff base for long-term controlled drug release. Int. J. Biol. Macromol..

[52] Mashaqbeh H., Obaidat R., Alsmadi M.M., Bardaweel S., Hailat N. (2024). Characterization and optimization of colon specific nanosponges immobilized polymeric microbeads formulation for the combined delivery of 5-fluorouracil and curcumin. J. Drug Deliv. Sci. Technol..

[53] Obaidat R., Kwiak A.D.A., Hamed R. (2022). Development of combined therapy of metronidazole and ibuprofen using in situ microgels for the treatment of periodontitis. J. Drug Deliv. Sci. Technol..

[54] Kolawole O.M., Lau W.M., Khutoryanskiy V.V. (2019). Chitosan/β-glycerophosphate in situ gelling mucoadhesive systems for intravesical delivery of mitomycin-C. Int. J. Pharm. X.

[55] Sadeghi D., Solouk A., Samadikuchaksaraei A., Seifalian A.M. (2021). Preparation of internally-crosslinked alginate microspheres: Optimization of process parameters and study of pH-responsive behaviors. Carbohydr. Polym..

[56] Essifi K., Lakrat M., Berraaouan D., Fauconnier M.-L., El Bachiri A., Tahani A. (2021). Optimization of gallic acid encapsulation in calcium alginate microbeads using Box-Behnken Experimental Design. Polym. Bull..

[57] Khataybeh B., Jaradat Z., Ababneh Q. (2023). Anti-bacterial, anti-biofilm and anti-quorum sensing activities of honey: A review. J. Ethnopharmacol..

[58] Zeng Q., Han Y., Li H., Chang J. (2015). Design of a thermosensitive bioglass/agarose–alginate composite hydrogel for chronic wound healing. J. Mater. Chem. B.

