# Supplementary material for: Hyaluronic Acid/Chitosan/Glycerophosphate-Based In Situ-Forming Hydrogel for Accelerated Wound Healing
Source: Gels. 2025 Oct 17;11(10):835. doi: 10.3390/gels11100835 (PMC12563622; doi:10.3390/gels11100835)
Supplement: Supplementary file 1 [file gels-11-00835-s001.zip › gels-3894079-supplementary.pdf]

Supplementary Information (SI)

Hyaluronic Acid/Chitosan/Glycerophosphate-Based In-Situ Forming Hydrogel for Accelerated Wound Healing

Hadeia Mashaqbeh [1], Rania Hamed [2], Hiba Alzoubi [3], Rana Obeidat [4], Mohammad Alnaeif [5], Meriem Rezigue [1], Hala T Abukassab [1], Wasan Al-Farhan[2] and Mohammad Obeid [1]

[1] Pharmaceutics and Pharmaceutical Technology Department, Faculty of Pharmacy, Yarmouk University, Irbid 21163, Jordan.
[2] Department of Pharmacy, Faculty of Pharmacy, Al-Zaytoonah University of Jordan, P.O. Box 130, Amman, 11733, Jordan
[3] Department of Diseases Basic Sciences, Faculty of Medicine, Yarmouk University, Irbid, Jordan
[4] Department of Pharmaceutics and Pharmaceutical Technology, Faculty of Pharmacy, The University of Jordan, Amman, Jordan
[5] Pharmaceutical and Chemical Engineering Department, School of Applied Medical Sciences, German Jordanian University, Amman Madaba Street, P.O. Box 35247, Amman, 11180, Jordan
* Correspondence: Department of Pharmaceutics and Pharmaceutical Technology, Faculty of Pharmacy, Yarmouk University, Irbid 21163, Jordan, P.O. BOX 566; Telephone: 0096227211111-7261; Email: h.mashaqbeh@yu.edu.jo, ORCID: 0000-0002-9376-2711

* Address correspondence to this author at:

Department of Pharmaceutics and Pharmaceutical Technology
Faculty of Pharmacy
Yarmouk University
Irbid 21163, Jordan, P.O. BOX 566
Telephone: 0096227211111-7261
Email: h.mashaqbeh@yu.edu.jo

Table S1: FTIR Assignment of raw Hyaluronic acid, Chitosan and Ciprofloxacin.

Wavenumber(cm^{-1})	Assignments	Compound	reference
**3450–3200**	O-H and N-H stretching (hydroxyl and amine groups)	Chitosan	[47]

**2880**	C-H stretching (alkyl groups)	Chitosan	6]
**1650**	C=O stretching (amide I)	Chitosan	[47, 48]
**1576**	Amide II vibrations	Chitosan	[48]
**1590**	N-H bending (Amide II band)	Chitosan	[47]
**1420**	C-H bending (CH_2 groups)	Chitosan	[48]
**1380**	C-H bending (CH_3 groups)	Chitosan	[47, 48]
**1020-1150**	alcohol C-O stretch and C-O-C stretching	Hyaluronic acid	[49-52]
**1450**	C-O-H bending of carboxylic acid	Hyaluronic acid	[49-52]
**1600-1620**	N-H bending and C-N stretching in the amide group (amide II) band	Hyaluronic acid	[49-52]
**2920-2990**	C-H methyl, symmetric stretch (polysaccharide signals)	Hyaluronic acid	[49-52]
**3300**	OH stretching, and N-H stretching vibrations in the N-acetyl side chain	Hyaluronic acid	[49-52]
**3500–3450**	O–H stretching vibrations	Ciprofloxacin	[53]
**3373**	N–H stretching vibrations	Ciprofloxacin	[53]
**1750–1700**	Carbonyl (C=O) stretching vibrations	Ciprofloxacin	[53]
**1623**	C=C stretching vibrations	Ciprofloxacin	[53]
**1342**	C–N stretching vibrations	Ciprofloxacin	[53]

**1267**	C–O stretching vibrations	Ciprofloxacin	[53]
**1050**	C-F groups	Ciprofloxacin	[53]
